# Exploring Potential Impact of Graphene Oxide and Graphene Oxide-Polyethylenimine on Biological Behavior of Human Amniotic Fluid-Derived Stem Cells

**DOI:** 10.3390/ijms252413598

**Published:** 2024-12-19

**Authors:** Andrea Di Credico, Giulia Gaggi, Sandra Bibbò, Serena Pilato, Samanta Moffa, Stefano Di Giacomo, Gabriella Siani, Antonella Fontana, Fani Konstantinidou, Marisa Donato, Liborio Stuppia, Valentina Gatta, Angela Di Baldassarre, Barbara Ghinassi

**Affiliations:** 1 Department of Medicine and Aging Sciences, “G. d’Annunzio” University of Chieti-Pescara, 66100 Chieti, Italy; andrea.dicredico@unich.it (A.D.C.); giulia.gaggi@unich.it (G.G.); sandra.bibbo@unich.it (S.B.); 2 Cell Reprogramming and Differentiation Lab, “G. d’Annunzio University” of Chieti-Pescara, 66100 Chieti, Italy; b.ghinassi@unich.it; 3 UdA-Tech Lab, “G. d’Annunzio” University of Chieti-Pescara, 66100 Chieti, Italy; serena.pilato@unich.it (S.P.); antonella.fontana@unich.it (A.F.); 4 Department of Pharmacy, “G. d’Annunzio” University of Chieti-Pescara, 66100 Chieti, Italy; samanta.moffa@unich.it (S.M.); stefano.digiacomo@unich.it (S.D.G.); gabriella.siani@unich.it (G.S.); 5 Department of Neuroscience, Imaging and Clinical Sciences, School of Medicine and Health Sciences, “G. d’Annunzio” University of Chieti-Pescara, 66100 Chieti, Italy; fani.konstantinidou@unich.it (F.K.); mdonato1@unite.it (M.D.); liborio.stuppia@unich.it (L.S.); valentina.gatta@unich.it (V.G.); 6 Unit of Molecular Genetics, Center for Advanced Studies and Technology (CAST), “G. d’Annunzio” University of Chieti-Pescara, 66100 Chieti, Italy; 7 Department of Innovative Technologies in Medicine and Dentistry, “G. d’Annunzio” University Chieti-Pescara, 66100 Chieti, Italy

**Keywords:** GO, GO-PEI, hAFSCs, cell proliferation, mitochondrial activity, miRNAs, scaffold, regenerative medicine, tissue engineering

## Abstract

Regenerative medicine and tissue engineering aim to restore or replace impaired organs and tissues using cell transplantation supported by scaffolds. Recently scientists are focusing on developing new biomaterials that optimize cellular attachment, migration, proliferation, and differentiation. Nanoparticles, such as graphene oxide (GO), have emerged as versatile materials due to their high surface-to-volume ratio and unique chemical properties, such as electrical conductivity and flexibility. However, GO faces challenges such as cytotoxicity at high concentrations, a negative surface charge, and potential inflammatory responses; for these reasons, variations in synthesis have been studied. A GO derivative, Graphene Oxide-Polyethylenimine (GO-PEI), shows controlled porosity and structural definition, potentially offering better support for cell growth. Human amniotic fluid stem cells (hAFSCs) are a promising candidate for regenerative medicine due to their ability to differentiate into mesodermic and ectodermic lineages, their non-immunogenic nature, and ease of isolation. This study investigates the effects of GO and GO-PEI on hAFSCs, focusing on the effects on adhesion, proliferation, and metabolic features. Results indicate that GO-PEI restores cell proliferation and mitochondrial activity to control levels, with respect to GO that appeared less biocompatible. Both materials also influence the miRNA cargo of hAFSC-derived microvesicles, potentially influencing also cell-to-cell communication.

## 1. Introduction

Regenerative medicine and tissue engineering are pioneering fields in life sciences, aiming to restore or replace lost or impaired organs and tissues through cell transplantation supported by scaffolds [1]. Recently, scientists have focused on developing new biomaterials that can provide optimized support for cellular attachment, migration, proliferation, and differentiation, helping to replace, regrow, or repair tissues [1,2]. Nanomedicines, which exploit nanomaterials ranging from 10 to 200 nm in size, offer significant multifunctionality due to the high surface-to-volume ratio and peculiar surface and chemical properties of nanoobjects. Various nanoscale structures, such as dendrimers, liposomes, polymer-based nanoparticles, micelles, carbon nanotubes, and graphene derivatives, were created for this purpose. In this scenario, graphene oxide (GO), produced by oxidizing graphene, has emerged as a versatile nanomaterial with extensive applications in biomedicine, particularly in the realm of regenerative medicine and tissue engineering. It supports the possibility to create a niche for stem cell growth that is crucial for stem cell biology since the microenvironment and substrate play a pivotal role in direct stem cell fate [3]. In addition, since it is a flexible substrate and can be easily transformed in an electrically conductive nanomaterial, it could be used to create a 3D scaffold for cardiac and muscle engineering or as a nerve guidance conduit for peripheral nerve regeneration [1,4]. It is generally preferred over graphene for creating homogeneous aqueous suspensions due to the oxygen-containing functional groups on its basal plane (hydroxyl and epoxide groups) and edges (carboxyl groups).

The interaction between GO and stem cells was the subject of numerous studies, revealing both promising results and notable challenges [5]. On one hand, GO has been shown to support stem cell adhesion, proliferation, and differentiation into various lineages such as osteogenic, neural, and adipogenic pathways. Its biocompatibility and the demonstrated [6] ability of small GO nanoflakes to interact with the cell membrane, mimicking extracellular mechanical signaling and promoting the release of microvesicles, further enhance its appeal as a biomaterial scaffold. However, the application of GO is not without limitations. Concerns regarding its cytotoxicity, long-term stability, and potential inflammatory responses highlight the need for a thorough understanding of its interactions with biological systems [7]. Additionally, variations in the synthesis and functionalization of GO can lead to inconsistent outcomes, complicating the reproducibility and reliability of experimental results [7]. A derivative of GO, Graphene Oxide-Polyethylenimine (GO-PEI) was created through the covalent functionalization of GO with linear polyethylenimine (PEI) macromolecules. Previous studies have shown that, although cross-linking between GO and PEI results in the formation of structurally defined tridimensional structures with controlled porosity, the most effective sample able to promote cell growth was the one layer of GO functionalized with PEI [8].

Human amniotic fluid stem cells (hAFSCs) are a population of stem cells that can be isolated from amniotic fluid during amniocentesis [9]. They were always considered multipotent and committed through the mesodermal lineage [2,10,11]. Despite that, recent studies have demonstrated that they share some epigenetic and genetic features with human-induced pluripotent stem cells (hiPSCs) [12] and can give rise to ectodermal lineages such as motor neurons [13]. These properties make hAFSCs very good candidates in the field of regenerative medicine, since they can be easily isolated during medical routine procedures without any ethical issues, have neither immunogenic nor tumorigenic potential, are less expensive, and are easier to culture than hiPSCs [12]. Anyway, to avoid altering the differentiation capacity of the cells, hAFSCs growing on scaffolds should retain all their biological and genetic features, including miRNA expression, since it was demonstrated that these non-coding RNAs are deeply implicated in the control of the developmental process and cellular differentiation [14].

This study aimed to investigate for the first time the effect of GO and GO-PEI on the adhesion, proliferation, and metabolic features of hAFSCs in order to identify the most suitable scaffold for regenerative medicine.

## 2. Results

### 2.1. GO and GO-PEI Precoated Coverslips Characterization

**Figure 1:**
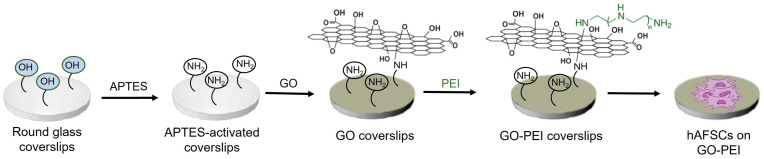
Representation of functionalization route of glass coverslips with GO and PEI and hAFSCs culture on GO-PEI substrates.

GO scaffolds were obtained by covalently linking GO to glass coverslips by exploiting the nucleophilic attack of 3-(aminopropyl)triethoxysilane (APTES) amine groups linked to the glass coverslips to the reactive epoxides on the GO flakes. Analogously, PEI was linked to GO scaffolds by nucleophilic attack of its free amine moieties with reactive groups in GO (Figure 1).

**Figure 2:**
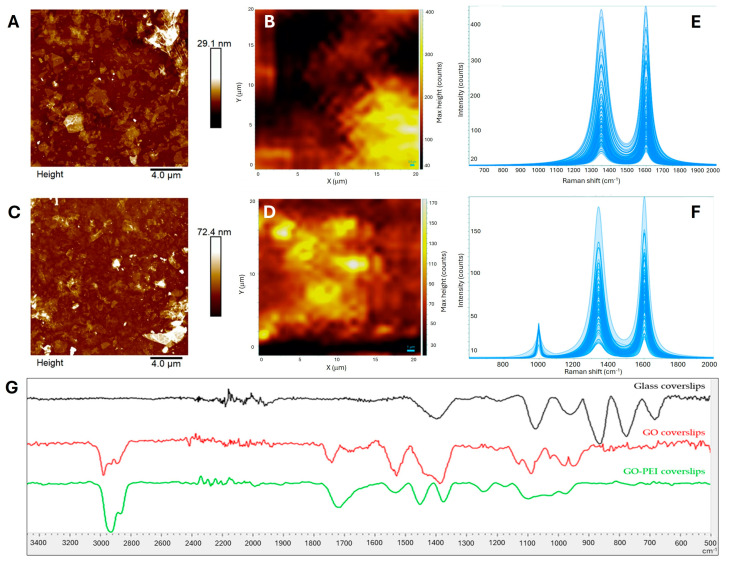
AFM images of surface topography and Raman mapping of (**A**,**B**) GO functionalized coverslip and (**C**,**D**) GO-PEI functionalized coverslip; (**E**) overlayed Raman spectra of 400 µm^2^ area of GO-coated glass coverslip; and (**F**) overlayed Raman spectra of 400 µm^2^ area of GO-PEI-coated glass coverslip. In AFM micrographs, color represents the height of deposited materials with height increasing from brown dots to white dots. In Raman mapping, intensity of G peak, corresponding to GO concentration, increases when passing from black to yellow regions. (**G**) FTIR spectra of glass coverslip (black line), GO-functionalized coverslip (red line), and GO-PEI coverslip (green line).

The obtained glass coverslips coated with GO and GO-PEI were characterized by using Atomic Force Microscopy (AFM) and Raman and IR spectroscopy. The AFM analysis revealed the presence of individual GO sheets well visualized onto the surface of the GO and GO-PEI functionalized coverslips. In both samples, it was possible to notice bright ripples due to the random reaction of the GO flakes with the APTES-activated surface and the consequent GO sheets overlapping. In addition, the bearing analysis tool of AFM software (Nanoscope Analysis 1.8, Bruker, Billerica, MA, USA) demonstrated a GO surface coverage of 44.3 (±1.4)% over a total surface area of 400 µm^2^, whereas the addition of PEI increases the coverage to 59.3 (±0.6)%, corresponding to 237.2 µm^2^ (Figure 2A,C).

To further confirm the distribution of the GO flakes on the coverslips surface, Raman spectroscopy analysis was performed. The Raman spectra of the GO-functionalized and GO-PEI-functionalized glass coverslips are reported in Figure 2E,F as overlayed deconvoluted spectra of a 400 µm^2^ area. The G peak (band at 1600 cm^−1^) is related to the graphitic-derived material breathing mode of C-C sp^2^, while the D peak (band at ≈1350 cm^−1^) refers to the defected graphene structure.

The Raman mappings, created by using the Raman peak intensity of GO deposited on the selected area, yield images of GO distribution and concentration. They are false color images in which each pixel is shaded according to the following: (i) the relative intensities of the graphene G band to evaluate the GO distribution (see Figure 2B,D); and (ii) the D bandwidth to identify the oxidation degree of the graphene oxide deposited on the glass coverslip (see Figure S1 in the Supplementary Materials). As shown in Figure 2B,D, Raman mappings demonstrated a complete coverage of GO on both samples despite the concentration of GO varying in the mapped area, with the concentration of GO increasing from the black regions to the yellow regions.

Raman spectroscopy is also used to evaluate the degree of GO functionalization with PEI. As is widely reported in the literature [15,16], the oxidation degree of GO, inversely proportional to the GO functionalization [8], is evaluated by analyzing the D peak width, with higher bandwidth indicating a more oxidized GO. In the GO-PEI functionalized substrate, a reduction in the oxidation degree, probably due to the covalent binding of PEI amine groups to graphene oxide epoxy moieties, can be inferred by comparing the D peak width of the GO samples before and after functionalization with PEI (compare Figure S1A,B in the Supplementary Materials, respectively).

As reported in Figure 2G, Figures S2 and S3 in the Supplementary Materials, IR spectra confirmed the occurred chemical functionalization of the glass coverslips. Firstly, the presence of graphene oxide in the GO-functionalized coverslips was highlighted by the peaks at around 1500–1600 cm^−1^ related to C=C in-plane vibrations and by the band above 1700 cm^−1^ due to the C=O stretching, as widely reported in the literature [17,18]. Furthermore, peaks at around 2800–3000 cm^−1^ were registered and related to the combination of C-H symmetric and asymmetric stretching vibrations of APTES and GO [19,20]. After the incubation of the GO coverslips into the PEI solution, the IR spectra showed differences in the 2900 cm^−1^ region, with an increased intensity of the C-H stretching bands due to the contribution of PEI carbonaceous skeletal chains [21]. The covalent functionalization between GO and PEI is confirmed by the new band at 1246 cm^−1^ due to C–N vibrations (bonded to an aromatic ring) [22] and the simultaneous decrease in the band at 1087 cm^−1^, which is attributable to C-O stretching vibration of the epoxide groups [22].

### 2.2. GO and GO-PEI Precoated Coverslips Influenced Cellular Adhesion and Proliferation

**Figure 3:**
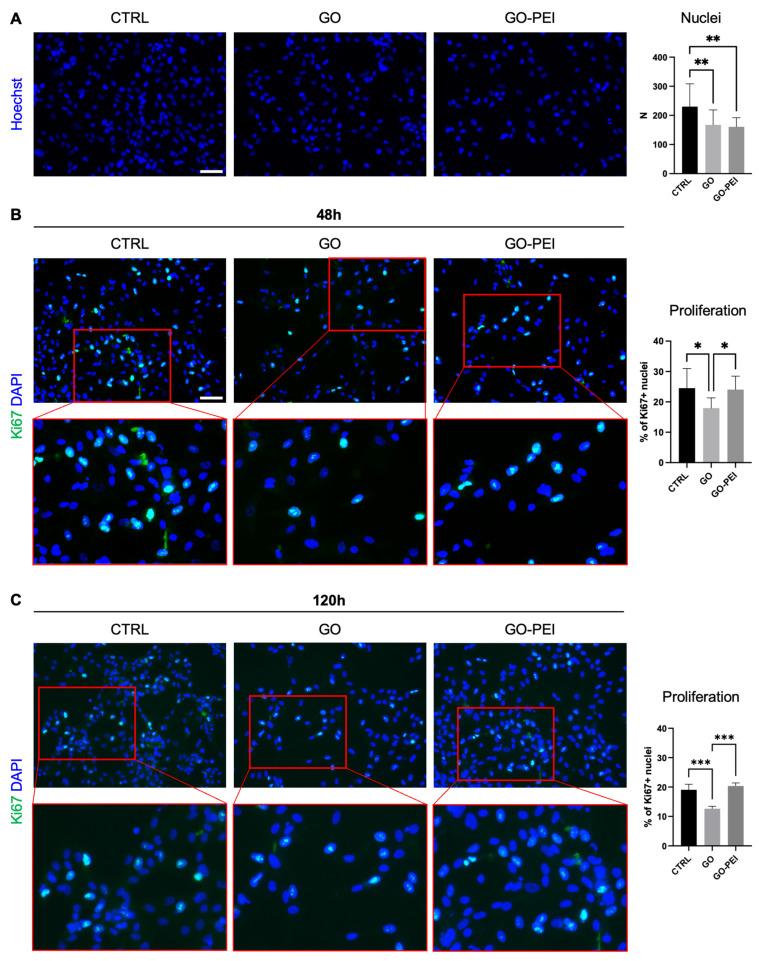
Effect of GO and GO-PEI on cellular adhesion and proliferation. (**A**) Cellular attachment on different types of supports, as indicated, was evaluated 24 h after seeding. Cells were fixed and nuclei were counterstained with Hoechst 33342 (blue). Magnification 20×, scale bar 100 µm. Histogram showed number of nuclei counted for each condition. Data expressed as mean ± SD. (**B**,**C**) Immunohistochemical detection of Ki67 (green fluorescence) at 48 h and 120 h after seeding, as indicated. Nuclei were counterstained with DAPI (blue). Magnification 20×, scale bar 100 µm. Histogram indicates % of Ki67^+^ nuclei in different experimental conditions, as indicated. Data expressed as mean ± SD. * *p* < 0.05, ** *p* < 0.01, *** *p* < 0.001.

In order to assess whether the GO and/or GO-PEI altered the cellular adhesion, hAFSCs were stained 24 h after seeding on different coated supports with Hoechst 33342. The cell count demonstrated a reduction in the attached cells on the GO and GO-PEI supports (Figure 3A), suggesting that these two derivatives of graphene interfere with cellular attachment. Despite that, when the cellular proliferation rate was analyzed by Ki67 expression, hAFSCs on GO-PEI supports displayed a proliferation rate comparable to the CTRL condition, whereas the GO condition showed a reduced percentage of Ki67^+^ cells, both at 48 h and 120 h (Figure 3B,C).

**Figure 4:**
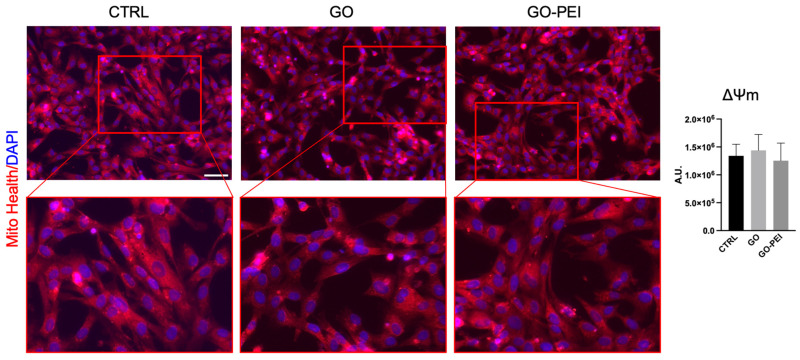
Effect of GO and GO-PEI on MMP. Detection of MMP (red fluorescence) 48 h after seeding for each condition. Nuclei were counterstained with DAPI (blue). Original magnification: 20×, scale bar 100 µm. Histogram indicates fluorescent intensity normalized by number of nuclei in each field. Data expressed as mean ± SD.

Then, MitoHealth, a specific red fluorescent probe that accumulates in healthy mitochondria in a proportional manner with mitochondrial membrane potential (MMP), was used to investigate whether BPs and PFs can harm mitochondrial activity. Data reported in Figure 4 showed that neither GO nor GO-PEI altered the MMP in hAFSCs.

### 2.3. GO-PEI Restores Expression of Mitochondrial Respiratory Chain Proteins in hAFSCs

Mitochondria plays a pivotal role in regulating cell biology cycle and proliferation [23]. Since the alterations of the cellular proliferation rate were detected in the cells growing on coverslips coated with GO, but unexpectedly no MMP modifications were reported, a deeper analysis of the expression protein complexes belonging to the mitochondrial oxidative phosphorylation system (OXPHOS) was performed.

**Figure 5:**
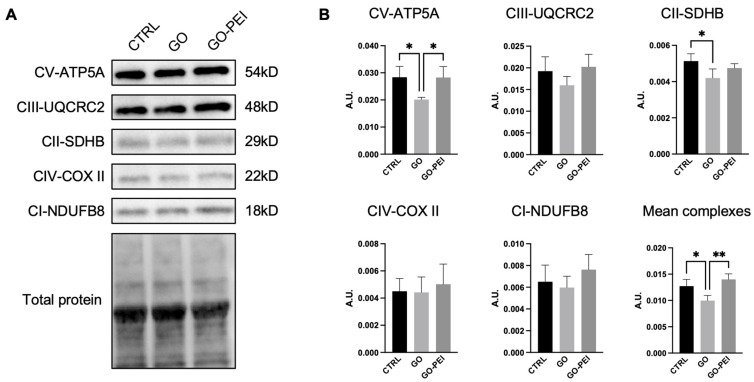
Protein expression of mitochondrial respiratory chain complexes. (**A**) Representative Western blot showing changes in expression of (OXPHOS) proteins 48 h from seeding in different conditions, as indicated. (**B**) Histograms show quantification of each protein, normalized on cellular total protein content. Data expressed as mean ± SD (*n* = 4) * *p* < 0.05, ** *p* < 0.01.

Data reported in Figure 5 showed that the hAFSCs growing for 48 h on GO-coated coverslips expressed lower levels of Complex II-Succinate dehydrogenase (CII-SDHB) and Complex V-ATP synthase (CV-ATP5A), while the protein expression of these two complexes was comparable in CTRL and GO-PEI samples, suggesting that this coating does not alter the mitochondrial functionality of stem cells. The difference in protein expression observed in the GO condition was also reflected in the mean complex expression (Figure 5B).

### 2.4. GO and GO-PEI Alter Secretion of miRNAs in hAFSCs

Since the secretion of microparticles (MPs) from stem cells is crucial as they play a significant role in intercellular communication and the modulation of the cellular environment, we evaluated whether the GO and GO-PEI substrates were able to affect the secretion of miRNAs from hAFSCs. Therefore, at 48 h after seeding, MPs were collected from the cells growing on the substrates and the miRNA content was analyzed. More specifically, the analyses focused on the expression of miRNAs involved in the regulation of cell proliferation, apoptosis, differentiation capacity, and mitochondrial functionality [24,25,26,27,28,29,30].

**Figure 6:**
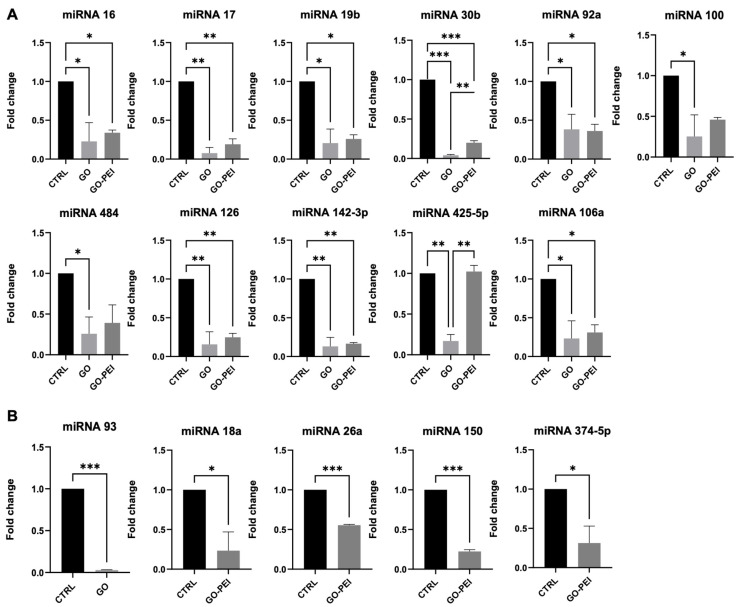
GO and GO-PEI substrates influence miRNA cargo of MPs released from hAFSCs. (**A**) miRNAs present in MPs from hAFSCs cultured in CTRL, GO, and GO-PEI; or (**B**) miRNAs present only in MPs from hAFSCs cultured on CTRL and GO or GO-PEI substrates. Fold changes were obtained using ΔΔCT method and normalized on CTRL condition. Data presented as mean ± SD of three independent experiments, * *p* ≤ 0.05, ** *p* ≤ 0.01, *** *p* ≤ 0.001.

The data showed a downregulation of miR-16, 17, 19b, 30b, 92a, 106a, 126, and 142-3p in both GO and GO-PEI with respect to the CTRL condition. On the other hand, miR-425-5p and miR-484 were significantly modulated only on GO supports (Figure 6A). More interestingly, miR-93, which has a role in neuronal progenitor cell proliferation, neuronal growth, and reduces inflammatory cytokines expression [31], was downregulated in GO and totally undetectable in the MPs from the GO-PEI condition. Similarly, miR-374-5p, 18a, 26a, and 150 were reduced to very low levels in GO-PEI and not detected in GO (Figure 5B). Moreover, miR-7e, 20a 24, 29a, 99a, 221 451, and 597 were not modulated either in GO nor in GO-PEI (Supplementary Figure S4). These data suggest that substrates for cell cultures can alter the cargo from MPs influencing cell-to-cell communication.

## 3. Discussion

Cell culture substrates play a pivotal role in influencing cell behavior, including adhesion, proliferation, differentiation, and overall functionality. Indeed, thanks to physical, chemical, and topographical properties, cell supports can significantly affect the interaction between the stem cells and the environment. A well-designed substrate can mimic the natural extracellular matrix, providing essential cues that guide stem cell fate and function [32]. For instance, surface chemistry can promote or inhibit cellular adhesion, while mechanical properties can influence differentiation [32]. The importance of substrate selection is underscored by studies showing that modifications such as functionalization with bioactive molecules can enhance cellular responses, leading to improved outcomes in tissue engineering and regenerative medicine [33]. Understanding and optimizing the interaction between stem cells and their substrates is therefore crucial for advancing stem cell-based therapies and achieving successful tissue regeneration [33]. In this scenario, graphene and GO have emerged as promising materials in stem cell research due to their unique physical and chemical properties, which can significantly influence stem cell behavior [34]. Graphene has exceptional strength and high electron conductivity, making it an attractive scaffold for supporting cell growth, the differentiation of nerve tissues, and cardiac tissue repair [34]. However, pure graphene has a hydrophobic nature and can hinder cellular attachment and proliferation. In contrast, GO, with its hydrophilic surface and abundant oxygen-containing functional groups, offers a better interaction with cells but can still present challenges such as reduced cell adhesion and potential cytotoxicity [35]. This highlights the necessity for further research to improve graphene-derived supports.

Despite the fact that previous studies have reported that the functionalization of GO with PEI can improve the biocompatibility of GO, making it a more attractive substrate for biological studies [8], its effects have never been investigated on the behavior of stem cells. Here, we focused on hAFSCs, fetal annexes-derived stem cells that can be considered a safer and cheaper alternative to hiPSCs for mesenchymal but also cardiac and neuronal differentiation [11,13]. In this scenario, a scaffold that helps the cells to maintain all their biological and stemness features, also supporting their differentiation, is becoming very attractive for the scientific community.

Here, for the first time, we demonstrated that GO-PEI (1) significantly favors hAFSC proliferation; (2) allows for the maintenance of a healthy mitochondrial respiratory chain, which is fundamental to maintain the integrity of their differentiation potential; and (3) can influence the miRNA content of MPs released from hAFSCs.

Cell proliferation plays a crucial role in regulating tissue homeostasis, repair, and development [36]. Our data clearly demonstrated that, despite both GO-PEI and GO reducing cellular adhesion at 24 h after seeding, GO-PEI favors the maintenance of a cellular proliferation rate comparable to the CTRL, whereas it was significantly decreased on the GO supports. Results obtained from the GO-PEI substrates are in line with the literature, showing that this coating does not alter cell viability and proliferation [37]. The effects of GO are more controversial, indeed some research groups reported that this coating inhibits the formation of glioblastoma spheroids or inhibited breast cancer cell growth [38,39], whereas Zheng et al. reported an increased proliferation of prostate cancer cells [40]. These differences could be due to the different GO concentration used in the studies, oxygen content and dimensions of GO flakes, different GO–cell interactions, and different biological features of cancer cell types. For example, Wang et al. used GO flakes of 0.2–22 µm in the culture medium [38], while Zheng et al. investigated much smaller 200–900 nm sized GO flakes [40]. In both studies, GO flakes were simply added at different concentrations in the culture medium rather than covalently bound to the glass substrate, as reported in the present study.

The reduced attachment of our hAFSCs on GO and GO-PEI substrates suggests that they may interfere with the initial cellular adhesion processes. Indeed, it was shown that graphene-based materials could affect the integrin signaling pathways that are fundamental for cell adhesion [41]. Despite that, GO-PEI seems to allow normal proliferation, potentially due to the probably positive charged PEI polymers of GO-PEI that interact favorably with the negatively charged cell membrane [42], improving the biocompatibility. Therefore, the surface properties of GO-PEI may create a more suitable environment for hAFSCs, supporting the maintenance of a good cell proliferation rate that is necessary for cellular expansion in many therapeutics applications and differentiation processes [43]. On the other hand, GO may create a less biocompatible environment that may alter the cell cycle progression and cytoskeleton structures that were already observed for graphene derivate materials [44]. Anyway, further analyses are required to elucidate in detail the molecular mechanism underlying the effects of GO and GO-PEI supports on cell adhesion and proliferation.

In stem cells, the maintenance of healthy mitochondria plays a pivotal role [45]. They are complex organelles essential for cellular energy production and the regulation of cellular pathways involved in proliferation, differentiation, cell cycle progression, and apoptosis [46]. Maintaining the integrity of MMP and the oxidative phosphorylation chain is crucial for preserving mitochondrial structure and function. Their disruption is associated with impaired cell proliferation and differentiation, stem cell aging, and cell death [47,48,49]. Moreover, it was demonstrated that glycolysis and OXPHOS are critical regulators of stem cells fates since they contribute to regulating both self-renewal and differentiation capacity [50]. More specifically, it was reported that stem cells have a metabolic transition from glycolysis to OXPHOS metabolism during the differentiation process, and OXPHOS inhibition leads to an impairment of differentiation capacity in embryonic stem cells [51,52]. Our data demonstrated that the GO-PEI substrate did not affect the expression of the mitochondrial respiratory complexes, whereas GO supports altered the expression of CII-SDHB and CV-ATP5A. In particular, the latter complex is responsible for the production of ATP [53] and the reduction in this molecule could lead to cell energy depletion that consequently may contribute to a decrease in cell proliferation rate and cause cell death [54]. Despite the reduced expression of the two OXPHOS complexes in cells growing on GO-coated supports, the HSC stain did not detect any changes in MMP, probably because CII-SDHB and CV-ATP5A are the only two mitochondrial respiratory complexes that are not involved in the formation of MMP [55,56]. Anyway, it was reported that the insufficient ATP synthase phosphorylating capacity can result in an impaired energy provision and an increased ROS production [57] that could lead to altered cellular biological functions and cell death. Our data on GO supports are also in line with Wierzbiki et al., who reported that muscle cell progenitors showed a reduced expression of CV-ATP5A, when cultured on a GO scaffold, with a potential impact on cell proliferation [58].

In addition, CII-SDHB and CV-ATP5A dysregulation might alter the differentiation potential of hAFSCs, especially through the cardiac and neuronal fates, since they require an increase in the OXPHOS metabolisms to begin commitment [59,60], but additional experiments are required to specifically address this point.

Very interestingly, the data demonstrated that functionalized supports were able also to influence the miRNA content in MPs released from hAFSCs. Our data showed that many miRNAs detected in MPs from hAFSC growing on GO and GO-PEI supports regulate the Pi3K-AKT pathway, cell proliferation, and mitochondrial functionality, but also neuronal differentiation [24,25,26,27,29,30,61,62,63]. More specifically, modulation of miR-425-5p and miR-484 in the GO condition aligns with the observed alteration in mitochondrial function since they are involved in regulating mitochondrial biogenesis and oxidative stress [28,64]. miR-93, which is reported to be upregulated in Parkinson’s disease (PD) [65], is downregulated or totally absent in the MPs from hAFSCs growing on GO and GO-PEI, respectively, suggesting a potential use of these extracellular vesicles in in vitro models of Parkinson’s disease to investigate their capacity of milder PD phenotypes. However, many miRNAs involved in the control of cell proliferation (miR-17, miR-106a, miR-30b, miR-92a [24,25,66,67]) were also downregulated in the GO-PEI condition, despite the fact that the Ki67 expression was comparable to the CTRL condition. This apparent discrepancy can be explained by the fact that miRNAs in MPs might not always correlate directly with the immediate cellular phenotype, considering that they are involved in cell-to-cell communication and can affect the neighboring cells in the microenvironment. Therefore, these data suggest that different coatings can influence hAFSCs through direct effects on cell behavior and indirect effects on recipient cells that can be located also far away by the alteration of the MP cargo content.

## 4. Materials and Methods

### 4.1. Characterization of GO and PEI Aqueous Solutions

The commercial aqueous solution of 4 g/L of graphene oxide from Graphenea (Donostia San Sebastian, Spain) was diluted with Ultrapure Milli-Q water (electric resistance > 18.2 MΩ/cm, Millipore Corp. model Direct-Q 3 system, Merck Millipore, Darmstadt, Germany) to reach the elected concentration of GO and bath-ultrasonicated for 30 min (37 kHz, 180 W; Elmasonic P60H; Elma Schmidbauer GmbH, Singen, Germany). The concentration of GO was spectrophotometrically checked at λmax of 230 nm by using a Varian Cary 100 BIO UV–vis spectrophotometer (Varian, Victoria, Australia). A linear polyethylenimine (PEI) aqueous solution was obtained by the solubilization of 8 mg of powder (average Mn 4000, PDI ≥ 1.3; MerkGaA, Darmstadt, Germany) in HCl-acidified Milli-Q water. The solution was stirred until clearing, neutralized with sodium hydroxide, and added to Milli-Q water to obtain a final concentration of 0.16 mg/mL.

### 4.2. GO and GO-PEI Preparation

Round glass coverslips were silanized by dipping the UV-ozone activated coverslips in 1 M 3-(aminopropyl)triethoxysilane (APTES) solution in ethanol (MerckGaA, Darmstadt, Germany). The silanized coverslips were then dipped in aqueous solutions of GO 20 μg/mL or sequentially in GO and PEI aqueous dispersions (in order to obtain GO or GO-PEI substrates, respectively) [8]. After incubation, the coverslips were rinsed with water and sterilized by UV irradiation. The surface topography and the distribution of GO flakes on the functionalized substrates were investigated by AFM and Raman spectroscopy mapping (Figure 2) analyses and then the substrates were used as support for amniotic fluid stem cell culture.

### 4.3. AFM Characterization of Functionalized Coverslips

The surface topography of the GO and GO-PEI functionalized coverslips was investigated with Atomic Force Microscopy (AFM) by using the MultiMode 8 AFM microscope (Bruker, Billerica, MA, USA) equipped with a Nanoscope V controller in Tapping Mode in air. A commercial silicon cantilever RTESPA-300 (rectangular geometry, resonance frequency 300 kHz and nominal spring constant 40 N/m) with a nominal tip radius of 8 nm was used to analyze the topography across a scan size of 20 µm × 20 µm. The images were analyzed by Nanoscope Analysis 1.8 software (Bruker, Billerica, MA, USA) and the bearing analysis tool was used to calculate the percentage of surface covered by GO flakes, in the form of both single layers and overlapped GO, and by the GO-PEI network.

### 4.4. ATR-FTIR, Raman Spectroscopy, and Raman Mapping of Functionalized Coverslips

IR spectra were collected using a Shimadzu IRAffinity-1S FTIR spectrophotometer (Shimadzu Italia S.r.l., Milan, Italy) equipped with an interferometer, a DLATGS (Deuterated Triglycine Sulfate Doped with l-Alanine) detector, and a single reflection diamond ATR crystal (QATR 10, Shimadzu Italia S.r.l., Milan, Italy). All FTIR spectra were recorded from 500 to 3500 cm^−1^ through the co-addition of 450 interferograms at a resolution of 4 cm^−1^ with Happ-Genzel apodization. The ATR crystal was carefully cleaned with acetone and ethanol before each analysis, a background was recorded for each sample using the glass coverslip, and the measurements were performed in triplicate. Spectra manipulation was performed with LabSolution IR version 2.27 (Shimadzu Italia S.r.l., Milan, Italy).

The Raman mapping of the GO-coated glass coverslips and the GO–PEI-coated glass coverslips (Figure 2) were obtained by confocal and a high-performance Raman microscope (XploRA PLUS, HORIBA, Kyoto, Japan) with deep-cooled CCD detector technology. LabSpec (HORIBA, Kyoto, Japan) was employed to control the Raman spectroscopic system and optimize and process the acquired data, to carry out Lorentzian deconvolution of the peaks. All Raman spectroscopic measurements were performed in the 600–2000 cm^−1^ range and with a 1800-line/mm grating. The samples were detected with a 532 nm laser, with a time of 1 s and 7 accumulations. Moderate power irradiation at the sample surface (∼10 mW) was used, focusing on the surface with a 100× objective to avoid laser-induced heating.

### 4.5. Cell Culture

Amniotic fluid samples were obtained after written informed consent in accordance with the Declaration of Helsinki. All samples had normal diploid karyotypes. The study was approved by the ethics committees and all experiments were performed in accordance with the relevant guidelines and regulations. The hAFSCs were isolated as previously described [14] from women undergoing amniocentesis for prenatal diagnosis at 16–17 weeks of pregnancy; the cells were cultured in Iscove’s modified Dulbecco’s medium (IMDM) (Thermo Fisher Scientific Waltham, MA, USA), supplemented with 20% fetal bovine serum (FBS, Corning New York, NY, USA), 1% penicillin/streptomycin, 2 mM l-glutamine, and 5 ng/mL basic fibroblast growth factor (bFGF, Thermo Fisher Scientific Waltham, MA, USA). The cells were cultured at 37 °C 5% CO_2_.

### 4.6. Mito Health Stain (HCS)

The mitochondrial functionality was analyzed by HCS Mitochondrial Health Kit (Thermo Fisher Scientific Waltham, MA, USA) following the manufacturer’s procedures. Briefly, hAFSCs were seeded at 1 × 10^5^ cell/well on the standard support (CTRL) or coverslip coated with GO and GO-PEI. After 48 h, the hAFSCs were incubated with MitoStain for 30 min at 37 °C and then fixed in 4% paraformaldehyde for 10 min. Nuclei were counterstained by Hoechst 33342. To quantify the fluorescence, 8 different fields for each sample were acquired by EVOS M7000 (Thermo Fisher Scientific Waltham, MA, USA) and analyzed by Celleste Image Analysis Software v5.0 (Thermo Fisher Scientific Waltham, MA, USA). The mean fluorescence intensity of each field was normalized on the number of the nuclei.

### 4.7. Cellular Attachment

Cellular attachment was determined by counting the number of nuclei. The hAFSCs were seeded at 1 × 10^5^ cell/well on the standard support (CTRL) or coverslip coated with GO and GO-PEI for 24 h. The cells were fixed in 4% paraformaldehyde for 10 min and the nuclei were counterstained by Hoechst 33342. The nuclei were automatically counted in 16 random fields for each condition by ImageJ v1.54k. The tool Watershed was used to discriminate singular nuclei within nuclear aggregates.

### 4.8. Immunofluorescence Analysis of Ki67 Staining

The hAFSCs were seeded at 1 × 10^5^ cell/well on the standard support (CTRL) or coverslip coated with GO and GO-PEI. After 48 h, immunofluorescence analysis was performed as previously described [68,69]. Briefly, the cells were fixed in 4% paraformaldehyde for 10 min and permeabilized by Triton 0.5% for 15 min. After blocking with 5% bovine serum albumin (BSA) for 30 min, the cells were stained with 1:100 anti-Ki67 Alexa fluor 488 conjugated antibody (Thermo Fisher Scientific Waltham, MA, USA) overnight at 4 °C. The nuclei were counterstained with 1:1000 DAPI (Thermo Fisher Scientific Waltham, MA, USA). Eight fields for each well were acquired by EVOS M7000 (Thermo Fisher Scientific Waltham, MA, USA) and analyzed by Celleste Image Analysis Software v5.0 (Thermo Fisher Scientific Waltham, MA, USA).

### 4.9. Western Blot (WB)

The hAFSCs were seeded at 1 × 10^5^ cell/well on the standard support (CTRL) or coverslip coated with GO and GO-PEI. After 48 h, the cells were lysed in RIPA Buffer (Thermo Fisher Scientific Waltham, MA, USA) supplemented with protease/phosphatase inhibitors (cOmplete/PhosSTOP™, Roche, Basilea, Switzerland). Protein quantification was determined in triplicate using the Rapid Gold BCA Protein Assay Kit (Thermo Fisher Scientific, Waltham, MA, USA) following the manufacturer’s instructions. For WB, 10 µg of the protein were loaded on 4–12% gradient precast gel (Biorad, Hercules, CA, USA). The proteins were then separated according to their molecular weight by SDS-PAGE and semi-dry transferred to a PVDF membrane (Millipore A/S, Copenhagen, Denmark) for 1 h. The membranes were blocked for 1 h in 5% BSA in tris-buffered saline (TBS) containing 0.1% Tween20 (TBST) and incubated in 1:1000 anti-OXPHOS (Abcam, Cambridge, MA, USA) at 4 °C overnight. The subsequent incubation in 1:1000 secondary antibody goat anti-mouse (Thermo Fisher Scientific, Waltham, MA, USA) was performed at room temperature for 1 h. The bands were visualized using Super Signal ECL (Thermo Fisher Scientific, Waltham, MA, USA) and acquired using iBright (Thermo Fisher Scientific, Waltham, MA, USA). Finally, the bands were quantified using ImageJ v1.54k and normalized as the total stain intensity obtained using the No-Stain™ Protein Labeling Reagent (Thermo Fisher Scientific, Waltham, MA, USA) following the manufacturer’s procedure.

### 4.10. RNA Extraction from MVs

Total RNA, including miRNAs, was isolated from all MVs by the Total Exosome RNA and Protein Isolation kit (Invitrogen, Thermo Fisher Scientific, Waltham, MA, USA). The quantity and quality of the total RNAs were assessed by the microvolume UV–vis spectrophotometer NanoPhotometer (Implen, GmbH, Munich, Germany).

### 4.11. MicroRNA Expression Profiling

MicroRNA expression analysis in MVs from the hAFSC culture on GO and GO + PEI substrates was carried out by the TaqMan™ Array Human MicroRNA A Cards v2.0 (Applied Biosystems, Foster City, CA, USA). A reverse transcription reaction was performed using the Megaplex RT primer Pool A and TaqMan MicroRNA Reverse Transcription kits (Applied Biosystems, Waltham, MA, USA). A preamplification reaction of complementary DNAs (cDNAs) was executed by the TaqMan PreAmp Master Mix 2X and Megaplex PreAmp Primers pool A (Applied Biosystems, Waltham, MA, USA). The preamplified products were diluted with 0,1X TE at a pH = 8.0 and mixed with the TaqMan™ Universal Master Mix II, no UNG (Applied Biosystems, Waltham, MA, USA), and nuclease-free water. They were then loaded into the TaqMan™ Array Human MicroRNA A plates following the manufacturer’s instructions and processed by qRT-PCR, using a QuantStudioTM 7 Pro Real-Time PCR detection system (Life Technologies, Carlsbad, CA, USA). Only miRNAs showing a maximum Ct = 35 and no outlier replicates were included in the analysis. In the TaqMan™ Array Human MicroRNA A Cards, three TaqMan MicroRNA Assay endogenous controls were included to aid in data normalization and one TaqMan MicroRNA Assay not related to human was included as a negative control.

### 4.12. Statistical Analysis

All data are presented as the mean ± SD of 3 independent experiments. The statistical analysis was performed by Prism 9 (GraphPad, San Diego, CA, USA) using the one-way ANOVA followed by Tukey’s post hoc. The level of significance was set at *p* < 0.05.

## 5. Conclusions

The present study revealed that GO-PEI coverslips promoted stem cell proliferation and growth, and restored OXPHOS metabolisms compared to GO substrates, which conversely poses some challenges on these cell functions. Overall, these findings underscore the importance of surface modification in influencing stem cell behavior and showed a higher biocompatibility of GO-PEI, since the alteration of proliferation and mitochondrial functionality could also impair the differentiation capacity of hAFSCs. These data may pave the way for the development of 3D GO-PEI scaffolds to potentially optimize stem cell proliferation and differentiation for applications in regenerative medicine and tissue engineering, especially for cardiac and neuronal differentiation. Indeed GO-PEI, displaying both the conductivity properties of graphene and the biocompatibility of PEI, may also improve and sustain the electrical properties of mature cardiomyocytes and neurons, paving the way to the generation of 3D transplantable scaffolds for in vivo tissue regeneration.

## Data Availability

The original contributions presented in this study are included in the article and Supplementary Materials. Further inquiries can be directed to the corresponding author.

## Supplementary Materials

The following supporting information can be downloaded at: https://www.mdpi.com/article/10.3390/ijms252413598/s1.
