# Supplementary material for: Exploring Potential Impact of Graphene Oxide and Graphene Oxide-Polyethylenimine on Biological Behavior of Human Amniotic Fluid-Derived Stem Cells
Source: Int J Mol Sci. 2024 Dec 19;25(24):13598. doi: 10.3390/ijms252413598 (PMC11678234; doi:10.3390/ijms252413598)
Supplement: Supplementary file 1 [file ijms-25-13598-s001.zip › ijms-3310989-supplementary.pdf]

Exploring the potential impact of Graphene Oxide and Graphene Oxide-Polyethylenimine on biological behavior of human amniotic fluid-derived stem cells

Andrea Di Credico[1,2,3†], Giulia Gaggi[1,2,3†], Sandra Bibbò[1,2,], Serena Pilato[3,4], Samanta Moffa[4], Stefano Di Giacomo[4], Gabriella Siani[4], Antonella Fontana[3,4], Fani Konstantinidou[5,6], Marisa Donato[5,6,7], Liborio Stuppia[5,6], Valentina Gatta[5,6], Angela Di Baldassarre[1,2,3*], Barbara Ghinassi[1,2,3]

[1] Department of Medicine and Aging Sciences, G. D'Annunzio University of Chieti-Pescara, 66100 Chieti, Italy
[2] Cell Reprogramming and Differentiation Lab, G. D'Annunzio University of Chieti-Pescara, 66100 Chieti, Italy
[3] UdA-Tech Lab, G. D'Annunzio University of Chieti-Pescara, 66100 Chieti, Italy
[4] Department of Pharmacy, G. D'Annunzio University of Chieti-Pescara, 66100 Chieti, Italy
[5] Department of Neuroscience, Imaging and Clinical Sciences, School of Medicine and Health Sciences, "G. d'Annunzio" University of Chieti-Pescara, 66100 Chieti, Italy
[6] Unit of Molecular Genetics, Center for Advanced Studies and Technology (CAST), "G. d'Annunzio" University of Chieti-Pescara, 66100 Chieti, Italy

[†] These authors contributed equally to this work
* Correspondence: a.dibaldassarre@unich.it

SUPPLEMENTARY MATERIAL

1. ***Raman spectroscopy***
2. ***ATR-FTIR spectroscopy***
3. ***miR Modulation***

1. Raman spectroscopy

Raman mapping was performed using also the D bandwidth to identify the oxidation degree of the graphene oxide deposited on the glass coverslip (Figure S1).

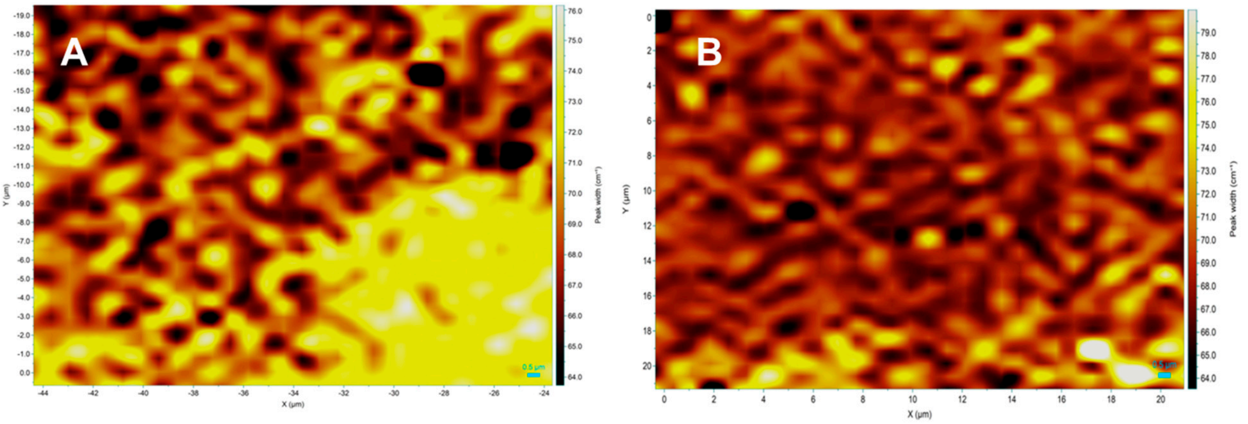

Figure S1. A) Raman mapping of D bandwidth of GO coated glass coverslip. B) Raman mapping of D bandwidth of GO-PEI coated glass coverslip. The colour of the maps refers to the high of the D bandwidth ranging from black regions, where the GO is less oxidized, to bright yellow regions, where the GO is more oxidized.

2. ATR-FTIR spectroscopy

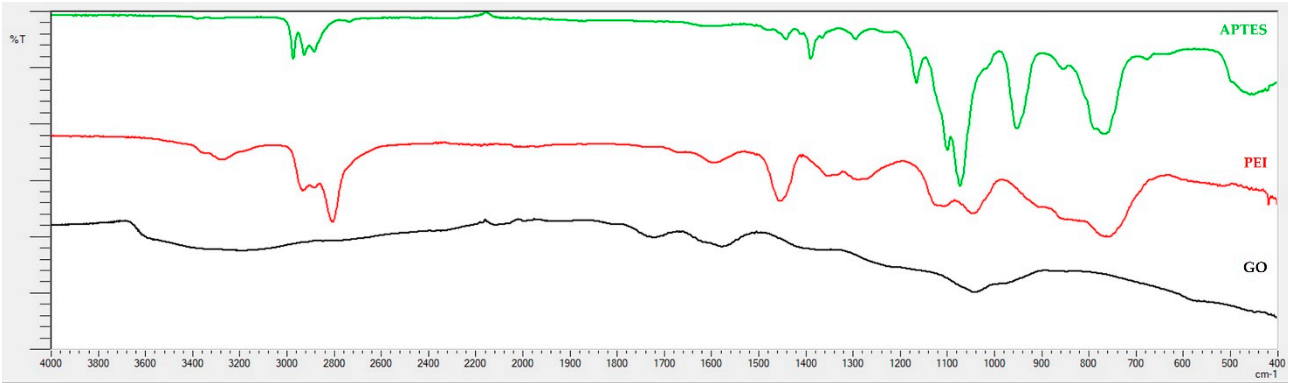

Figure S2. FTIR spectra of APTES (green line), PEI (red line) and GO (black line).

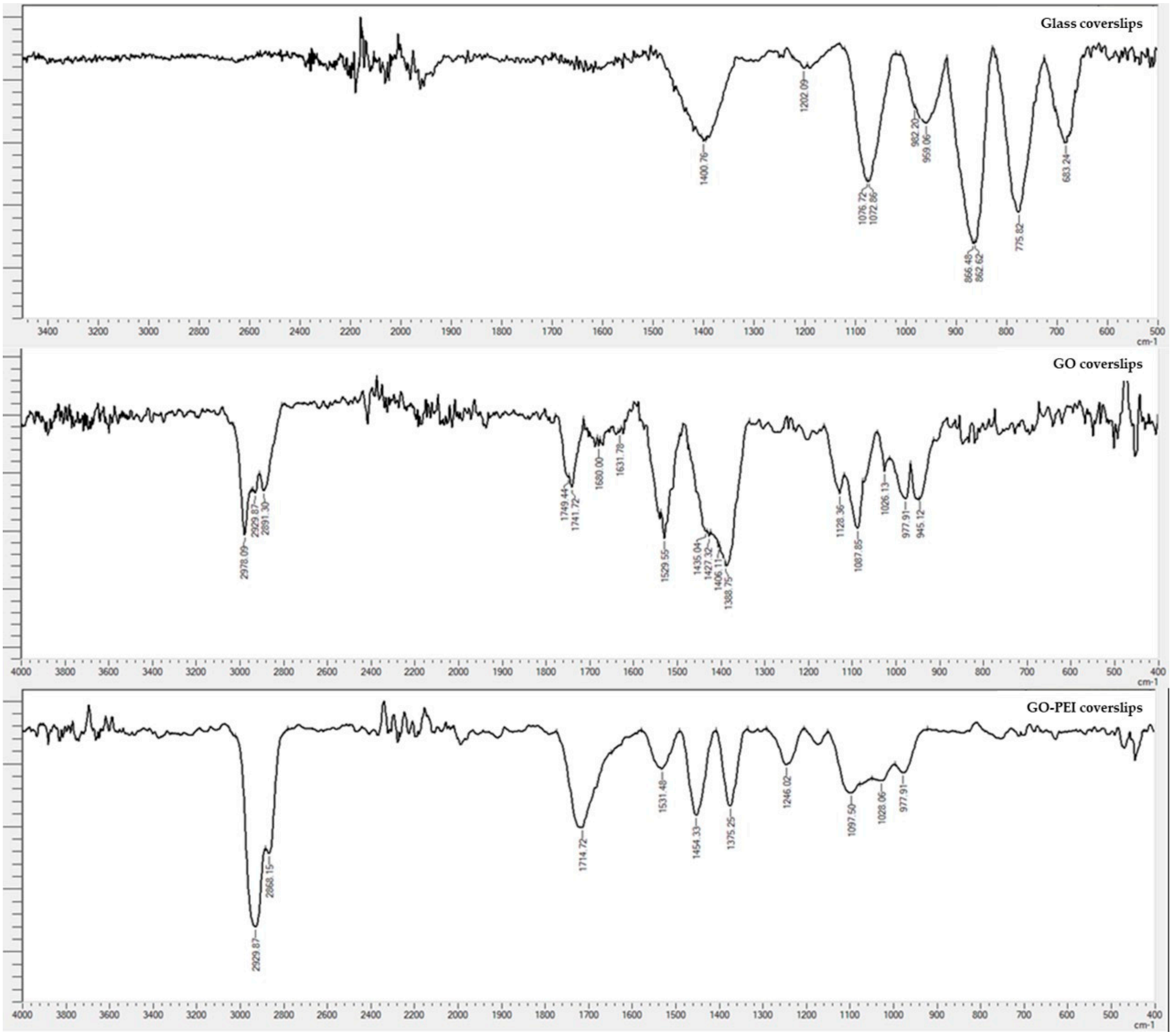

Figure S3. FTIR spectra of empty, GO and GO-PEI coverslips with relative peaks.

3. ***miR Modulation***

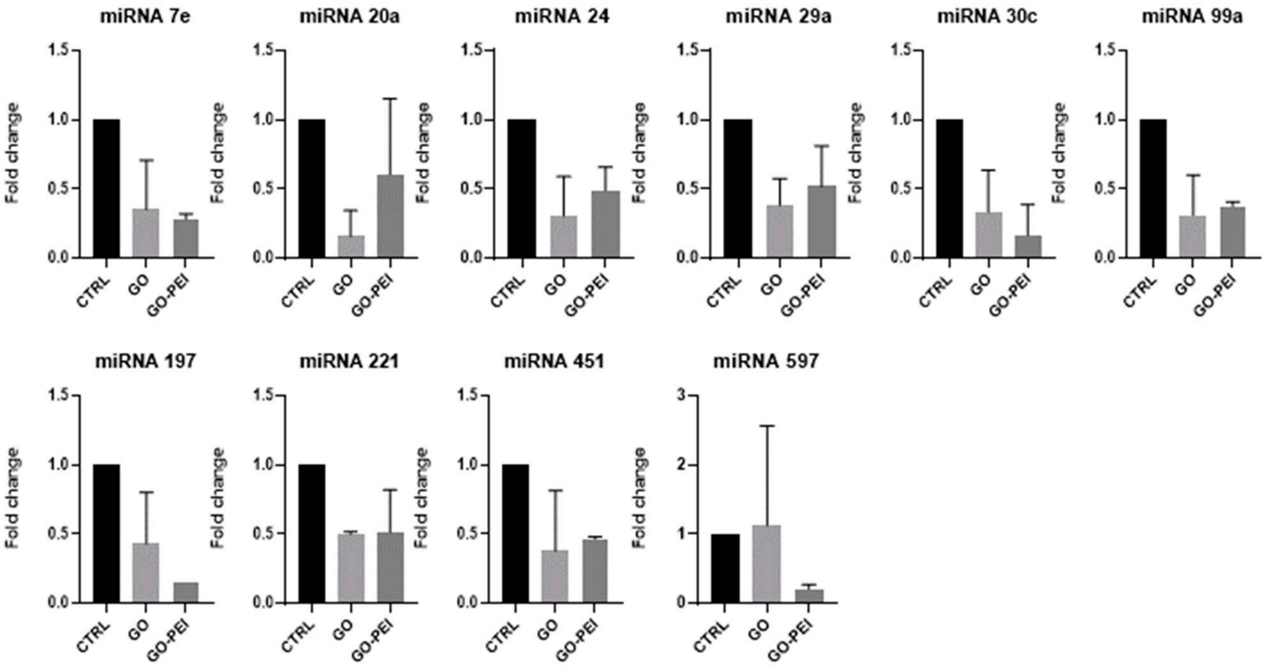

Figure S4. miRNA not modulated in cells growing on GO and GO-PEI compared to control condition (CTRL). miRNA expression was analyzed using TaqMan™ Array Human MicroRNA A.
